# Practical Methods of Anesthesia

**Published:** 1918-04

**Authors:** 


### ANESTHESIA

### *Practical Methods of Anesthesia.* By James T. Gwathmey, Captain, M. R. C., Abs. from the *Brit. Med. Jour.*, March 24, 1917.

The writer pleads for a more discriminating use of the word "practical" as applied to anesthesia. He considers an anesthetic method practical only when it fulfils the following conditions: adaptability to the patient, agreeableness in administration, maintenance of the patient in a condition as nearly normal as possible, and transition without pain or nausea.

Although all of these conditions can be met by modern methods, many of the best surgeons are inclined to entrust anesthesia to a nurse, who administers anesthetics in what the surgeon considers a "practical" manner with little regard to the requirements mentioned above. The administration of anesthetics is so important a factor in any operation that it should be entrusted to a specially trained anesthetist.

*Preliminary and Post-operative Medication.* — To forestall acidosis, sodium bicarbonate and lactose (1 drachm each) should be given every four hours for at least 48 hours before the operation. After the operation glucose should be administered by the rectum. Bonnot and Cleveland recommend quinine muriate, grains 10, dissolved in 2 ounces of water at 100° F., to be given immediately after operation, and to be followed in 30 minutes by saline proctoclysis, or, in septic cases, by six ounces of olive oil. From four to six doses are given at intervals of six hours. It is claimed that this treatment eliminates backache, and greatly reduces gas pains.

A preliminary narcotic treatment is indicated in nearly every case. This avoids the rise in blood pressure at the thought of operation and other changes that may interfere with the success of the anesthetic. Since morphine often causes nausea, the writer prefers :

| | |
|---|---|
| Paraldehyde . . . . . . . . . . . . . }<br>Pot. bromid. . . . . . . . . . . . . } | āā 4-8 grammes. |
| Aquae . . . . . . . . . . . . . . . . | to 120 cc. |

(Administered per rectum (Sim's position) 40 to 90 minutes before operation.).

By this procedure anesthesia is produced more quickly and with less ether, and hence with greater safety. It may possibly help to regulate acidosis, as Crile thinks morphine and bromides may do.

*Anesthetic Agents.* — In discussing the various anesthetics, the writer remarks that morphine when combined with alcohol is safer than when either of these drugs is used alone in emergency work for producing general anesthesia. Chloroform, anesthol, and ethyl chloride are useful as initiatory agents or to supplement other anesthetics.

He considers essence of orange (25 o/o oil of bitter orange peel and 75 o/o absolute alcohol), although not an anesthetic itself, as a safer and better introductory agent to ether by the closed method than nitrous oxide. Its odor is ten times as penetrating as ether vapor and it avoids the reflex stimulation by anesthetics of certain sensory nerves, which stimulation may interfere with respiratory mechanism and blood pressure. Oil of orange so reduces the sense of smell as to prevent all such reflex stimulation. It may also be used with good effect when ethyl chloride, chloroform, or anesthol are used as agents introductory to ether.

When chloroform is given with the proper apparatus providing for oxygen, air, and rebreathing throughout, the patient goes under and comes out of the anesthesia as quickly as with nitrous oxide and oxygen, and with immunity unimpaired. In emergency practice in administering chloroform, the mask should be surrounded with paper or a towel, and rebreathing permitted throughout. It is best to dip the bottle of chloroform from time to time into hot water, as heated anesthetics are safer than cold.

The writer condemns the " open drop ether " method as crude and unscientific. Much of the condemnation of ether at present is due to this faulty method of administration, for it increases the likelihood of vomiting, of anesthesia acidosis, and of resultant shock.

He considers the administration of nitrous oxide followed by ether as even a cruder and more dangerous method, for it raises the blood pressure and cyanoses the patient. The nitrous oxide-oxygen-ether sequence by the closed method removes all the objectionable features of the gas-ether sequence, and should be used whenever a closed method is indicated.

The writer describes the vapor mask which has enabled him to provide his patients as much " luxury of breathing " as results from the endotracheal or endopharyngeal methods. It is any chloroform mask covered with gauze and provided with some suitable arrangement for admitting warmed oxygen-ether vapor. If the vapor is passed to the mask, the method is called " open " or " semi-open " (the mask is usually covered with towels). If the vapor goes to a rubber bag, the method is called " closed ".

Oil-ether colonic anesthesia the writer prefers to any other method of colonic anesthesia.

He states that he uses a semi-open method of administering nitrous oxide-oxygen and warmed ether vapor. It is also oxygenated and deodorized. Thus the patient neither tastes nor smells the ether at any time. There is no strain upon the respiratory center.

He urges that the preliminary and after-treatment of the patient be left entirely to the anesthetist.

